# Control of Origin of Sesame Oil from Various Countries by Stable Isotope Analysis and DNA Based Markers—A Pilot Study

**DOI:** 10.1371/journal.pone.0123020

**Published:** 2015-04-01

**Authors:** Micha Horacek, Karin Hansel-Hohl, Kornel Burg, Gerhard Soja, Walter Okello-Anyanga, Silvia Fluch

**Affiliations:** 1 AIT- Austrian Institute of Technology GmbH, Health & Environment Department, 3430, Tulln, Austria; 2 Department of Agricultural Production, School of Agricultural Sciences, Makerere University, Kampala, P.O. Box. 7062, Kampala, Uganda, and National Semi-Arid Resources Research Institute (NaSARRI), Serere (Soroti), Uganda; Scottish Association for Marine Science, UNITED KINGDOM

## Abstract

The indication of origin of sesame seeds and sesame oil is one of the important factors influencing its price, as it is produced in many regions worldwide and certain provenances are especially sought after. We joined stable carbon and hydrogen isotope analysis with DNA based molecular marker analysis to study their combined potential for the discrimination of different origins of sesame seeds. For the stable carbon and hydrogen isotope data a positive correlation between both isotope parameters was observed, indicating a dominant combined influence of climate and water availability. This enabled discrimination between sesame samples from tropical and subtropical/moderate climatic provenances. Carbon isotope values also showed differences between oil from black and white sesame seeds from identical locations, indicating higher water use efficiency of plants producing black seeds. DNA based markers gave independent evidence for geographic variation as well as provided information on the genetic relatedness of the investigated samples. Depending on the differences in ambient environmental conditions and in the genotypic fingerprint, a combination of both analytical methods is a very powerful tool to assess the declared geographic origin. To our knowledge this is the first paper on food authenticity combining the stable isotope analysis of bio-elements with DNA based markers and their combined statistical analysis.

## Introduction

Sesame seed oil is a highly esteemed edible oil that is either consumed as edible oil, or used for pharmaceutical and health purposes. Sesame (*Sesamum indicum* L) is a very drought-tolerant plant, partly due to its extensive root system and also due to its high temperature tolerance. It is grown in many regions of the world [1]. Upon ripening, the sesame capsule splits open and releases the seed, therefore usually the plants are collected before ripening and stored vertically to prevent seed loss when the capsules split. Because of this shattering characteristic accompanied by its indeterminate growth, sesame is cultivated primarily on small plots that are harvested by hand. Due to this laborious harvesting sesame seeds are rarely produced in highly industrialized western countries. Around 80% of the world production of over 3.2 million tons are coming from the main sesame growing countries: India, China, Myanmar, Sudan, Uganda, Nigeria, Ethiopia, Pakistan, and Bangladesh (FAO, http://faostat.fao.org/site/567/). The discovery of an in-dehiscent (non-shattering) mutant by Langham in 1943 allowed breeding of high yielding, shatter-resistant varieties being used in industrialized countries like the USA [1].

In many countries local native products are favored to foreign ones, as has been investigated in Korea with respect to beef [2]. Thus, methods to control the declared origin of food products to protect regional brands and enhance consumer confidence are increasingly gaining importance. Since sesame seed oil of declared geographic origin is more highly valued by consumers than sesame oil without geographic designation, there is the danger of consumer deception by labeling or admixing cheaper sesame oil of a different geographic origin than indicated on the label to increase profit. Consequently methods are needed to control the declared origin of a produce and to identify the origin of sesame oil and seeds.

There are already numerous publications on the authenticity of sesame oil with respect to adulteration [3–9], however, there are almost no studies on the geographic origin or isotopic data of sesame oil available. Analyses and control of geographic origin of food stuff are routinely applied for wine in the EU, and data are stored in the EU—wine database [e.g. 10]. For many commodities such as meat, honey, cereals, vegetables and fruits several studies on using isotope analysis to proof geographic origin have been performed [e.g. 11–15, 42]. Regarding the geographic authenticity of vegetable oils, few studies applying stable isotopes have already been published, mainly about olive oil [e.g. 6, 16–18, 41]. For olive oil, also DNA based methods have been applied to test for adulteration [19, 20].

Stable isotope signals of plant material are influenced and controlled by the environmental conditions at the respective localities, thus isotope patterns of the samples are typical for the region they were growing in. Differences in isotopic patterns of samples from different origins therefore have the potential for the verification or control of geographic authenticity.

Carbon isotope values in plants are primarily influenced by the type of photosynthetic pathway (C_3_ or C_4_) and photosynthetic activity [21] as well as by the ambient environment. Therefore, climate and water availability are important factors, as water deficiency leads to the closure of the plant stomata to achieve a reduction of transpiration. This process also cuts the CO_2_ supply from the ambient air in the leaves and the plant has to utilize the CO_2_ gas trapped in the leaf interior. This results in a reduced discrimination of the heavy C-isotopes, as compared to a situation without CO_2_ limitation where heavy isotopes are less likely to be incorporated in newly synthetized carbohydrates [22].

Hydrogen isotopes in plants are dominantly influenced by the isotopic composition of the water being taken up by the plants [23]. Hydrogen and oxygen isotope signatures in precipitation water are globally influenced by two major processes: I) Isotope fractionation due to the ambient temperature (temperature effect) and II) isotope fractionation due to the continental effect (i.e. distance from the sea). The first process describes the preferred evaporation of water depleted in heavy isotopes at low temperatures resulting in precipitation with low isotope ratios at moderate to low temperature regions and precipitation enriched in heavy isotopes in warm and tropical regions. The second process explains the depletion in heavy isotopes in precipitation in inland areas compared to regions close to the sea due to the preferred out-raining of heavy isotopes, which passively enriches the remaining water vapor moving further inland in light isotopes. The described processes lead to precipitation globally enriched in heavy hydrogen and oxygen isotopes (^2^H and ^18^O) at low latitudes, low altitudes and close to the coast, and precipitation globally enriched in light hydrogen and oxygen isotopes (^1^H and ^16^O) at high latitudes, high altitudes and far away from the sea [24].

Besides stable isotope analysis, we also investigated the applicability of different DNA based molecular marker systems to assess geographic origin of sesame. A plant possesses different compartments within a cell, some of them holding their own genetic information, such as chloroplasts (cp), mitochondria (mt), nucleus(n). Depending on the compartment of the cell, the respective DNA entity shows different modes of inheritance (biparental [n], uniparental [cp, mt]), thereby allowing to answer different scientific questions. These range from genetic population diversity analysis using nuclear DNA to the assessment of phylo-geographic origin by cpDNA markers. With respect to traceability and origin, DNA based methods have been applied for the traceability of products from long-lived organisms like trees i.e. to trace the origin of tropical timber [25, 26] and to discriminate between accessions of apricot [27] or oak [28]. For sesame, so far only general molecular diversity analyses have been performed in collections from different countries such as Sudan [29], India [30], Turkey [31] or Vietnam and Cambodia [32] in order to determine genetic relations between germ plasm lines, or to estimate genetic diversity present [33–34].

In the present pilot study, we investigate sesame seed samples of different origins, and oil derived from them. Analysis of their stable carbon and hydrogen isotope signatures are applied, to assess if these elements can help to discriminate between oil samples of different provenances. Additionally the samples are investigated using DNA based molecular information as a possible complementary proxy. This is to our knowledge the first study on food authenticity combining molecular and bio-elements stable isotope investigations.

## Materials and Methods

Thirty eight different samples of sesame seed have been investigated (see Table 1). 21 of these with labeled origin from different localities in India (2), Egypt (4), Ethiopia (1), Mocambique (1), Turkey (1), Paraguay (5), El Salvador (2), Nicaragua (2), Bolivia (2) and Senegal (1) have been collected from producers and merchants presenting their goods at food fairs in 2009 and 2010 in Germany. Twelve samples were provided by collaboration partners of the National Semi-Arid Resources Research Institute (NaSARRI), Serere (Soroti), Uganda, and 5 originated from ICRISAT (International Crops Research Institute for the Semi-Arid Tropics), Nairobi, Kenya, where they have been grown. (Table 1). These latter samples from Uganda and Kenya were provided as bulked seed samples from single breeding lines from the breeding program of NaSARRI and ICRISAT.

**Table 1:** List of investigated sesame samples with county of origin.

sample	country of origin	d2H	d13C
**159684**	**Egypt**	-**112,7**	-**28,17**
**159685**	**Egypt**	-**100,3**	-**29,49**
**159686**	**Paraguay**	-**156,69**	-**28,51**
**159687**	**Paraguay**	-**154,45**	-**28,43**
**159688**	**Paraguay**	-**155,65**	-**28,49**
**159689**	**Paraguay**	-**157,87**	-**28,02**
**159690**	**Paraguay**	-**154,22**	-**28,07**
**159691**	**Turkey**	-**109,44**	-**27,59**
**159692**	**India**	-**120,2**	-**28,59**
**159693**	**unknown**	-**124,9**	-**28,44**
**161142**	**Egypt (d)**	-**129,99**	-**29,09**
**161143**	**Egypt**	-**129,09**	-**28,33**
**161144**	**Ethiopia**	-**136,02**	-**27,49**
**161145**	**El Salvador (d)**	-**168,75**	-**29,64**
**161146**	**El Salvador**	-**168,71**	-**28,65**
**161147**	**Senegal**	-**172,43**	-**29,6**
**161148**	**Bolivia**	-**194,14**	-**29,94**
**161149**	**Bolivia (d)**	-**214,91**	-**30,71**
**161150**	**Nicaragua**	-**175,53**	-**28,61**
**161151**	**Nicaragua (d)**	-**173,97**	-**29,72**
**161152**	**Mozambique**	-**157,63**	-**26,52**
**OTIS**	**Uganda**	-**123,7**	-**25,93**
**19**	**Kenya**	-**133,78**	-**28,09**
**SESIM 2**	**Uganda**	-**110,86**	-**26,25**
**20**	**Kenya**	-**131,73**	-**28,04**
(SESIM2*5181)-2-2-1	**Uganda**	-**117,27**	-**27,02**
(Local 158*6022–1)-2-1	**Uganda**	-**124,5**	-**25,9**
**U1-7**	**Uganda**	-**104,1**	-**24,49**
**Ajimo A1-5**	**Uganda**	-**105,19**	-**26,02**
**SESIM 1**	**Uganda**	-**109,43**	-**26,19**
**18**	**Kenya**	-**129,47**	-**28,4**
**ADONG 4–4**	**Uganda**	-**107,29**	-**25,4**
**LOCAL 158**	**Uganda**	-**113,4**	-**26,57**
Ajimo A1-6*7029–1	**Uganda**	-**112,07**	-**26,55**
**EM15-3-2**	**Uganda**	-**113,06**	-**26,13**
**AD-1-1-1**	**Uganda**	-**107,75**	-**24,23**

A (d) marks dark sesame seed samples.

For stable isotope analysis of the sesame oil, seeds of the different samples have been ground and an aliquot of 2 grams has been defatted using a soxhlet vacuum distillation unit with petrol ether (as previously described [35]. After the control for absence of solvent by constant weight 0.1–0.2mg of the extracted oil samples were weighed in silver and tin capsules for hydrogen and carbon isotope measurements, respectively. Hydrogen isotope analysis was performed with an elemental analyzer thermal combustion unit (TC/EA) connected via a ConFlo III to a Delta Plus XP (Finnigan) isotope ratio mass spectrometer (IRMS). For carbon isotope analysis the samples were introduced into a Carlo Erba (Finnigan) elemental analyzer (EA) connected to a MAT 251 (Finnigan) isotope ratio mass spectrometer (IRMS). Measurements were done at least in duplicate for C and N and at least in triplicate for H isotopes. Measurements are reported in the conventional δ notation with respect to the international standards VPDB (Vienna PeeDee Belemenite) and VSMOW (Vienna Standard Mean Ocean Water) for C and H isotopes, respectively. Reproducibility for δ^2^H is better than 3‰ and for δ^13^C better than 0.15‰ (1σ).

For the DNA based molecular analysis, DNA was extracted either from bulked ground seed material after oil extraction (21 probes from producers and merchants) or from single seeds after germination (samples from NASARRI). From each NASARRI seed lot seeds were germinated at 37°C for two days and afterwards further grown at room temperature for 3 days. 8 individual seedlings per seed lot were taken for separate genomic DNA extraction using DNeasy Plant Mini Kit (Qiagen), resulting in 136 individual DNA samples of the 17 different breeding lines. From the 21 bulked samples, genomic DNA was extracted using 50mg of ground material. 1 chloroplast (cp) and 9 nuclear microsatellite regions (SSR) were used to investigate genetic differences between the 38 regional samples. 6 nuclear SSR markers were selected from Dixit et al (2005) [33] and in addition, 8 EST (expressed sequence tag)-SSRs (Table 2) were developed based on ESTs available in the NCBI (National Center for Biotechnology Information; http://www.ncbi.nlm.nih.gov/) nucleotide database. From both sets a total of 9 nuclear SSR primer pairs were used for further investigations (CL93Contig1, GBssr_sa_72, BU670685, CL78Contig1, GBssr_sa_184, GBssr_sa_123, BU670267, GBssr_sa_108, Sesame09) as they proved to be variable in the investigated set of samples. From the 9 cpSSRs tested [36], only one cpSSR locus (ccmp2) proved to be variable.

**Table 2:** List of EST SSR containing fragments and the SSR motif they contain; sequences used for SSR identification originating from NCBI_DB_EST.

Name	Contig_Size	Primer sequences (5´-3´)		Repeat Motif	Tm
BU670267	singleton	Fwd*	GCAACTCCAGTCCCAGTCTC	(GA)4	61,4
		Rev	TCCATGGCGTTTGATGAGTA		55,3
BU670685	singleton	Fwd*	CCCAGCCAAGAAACAAGAAA	(TC)9, (AT)4	55,3
		Rev	AACCCCACTAGGCGAAGAAT		57,3
CL297Contig1	2	Fwd*	CGAGAAGGAGAACGATTTGG	(AT)6	57,3
		Rev	GTAACACTTCCGTCGCCATT		57,3
CL93Contig1	5	Fwd*	CAAAGCAAGAGACAAGATGACG	(CCG)4	58,4
		Rev	GTGGTGTTTGGACAGTGGTG		59,4
CL78Contig1	5	Fwd*	GAGCTCAGTGAGGAGAAAGCA	(TG)4	59,8
		Rev	AAGATGAAAGCAGGCAGCTC		57,3
CL569Contig1	2	Fwd*	CAGCTGGCAGATCAGTATGG	(GCA)4	59,4
		Rev	GCCCCTTCTTCTTCCTTGTT		57,3

All primer pairs listed above were tested in PCR using the ‘M13 tail PCR method’ [43], which involves three primers—two locus specific primers, and one universal primer tail for labelling. The sequence-specific forward primer has the M13 (-21) tail at its 5´end (5´ TGT AAA ACG ACG GCC AGT 3´) whereas the complementary universal M13 (-21) primer (5´ FAM-TGT AAA ACG ACG GCC AGT 3´) carries the fluorescent-label used for subsequent detection. Labelled PCR products were visualized on ABI PRISM 3130xl DNA sequencer (Applied Biosystems, AB) using Genemapper 4.0 software (AB) and sized against Rox 350 (AB).

Due to the fact that we were partially dealing with bulked samples, more than two alleles could be detected per sample per locus. For data analysis this meant that we had to treat all data as if they were stemming from bulked material, even for the samples from Uganda and Kenya, where single seed genetic information was available. Allelic data were scored like dominant markers, meaning that the presence of specific allele sizes was scored as 1, whenever this particular size was missing, the value was set to zero. For single seed analysis, allelic information was ‘pooled in silico’ to generate one data string per accession. This spread sheet could subsequently be used for principle component analysis of the DNA based information using GenAlEx 6.4 for. xls (http://biology.anu.edu.au/GenAlEx/Welcome.html).

Statistical analysis of the correlation between the two datasets was performed using the vegan R package [44–45]. A pseudo F statistics was calculated for both datasets by using the PERMANOVA approach [46]. Additionally the direct comparison between both datasets was performed by using a procrustes analysis (PROTEST) [47] and a mantle test [48] using R.

## Results

### Isotope data (Table 1, Fig 1)

Hydrogen and carbon isotope values of sesame oil samples from Uganda range from -125 to -104‰ VSMOW and from -27,0 to -24,2‰ VPDB, respectively and—134 to -130‰ VSMOW and—29,0 to -28,0‰ VPDB, respectively, for the Kenyan samples. Indian sesame oil samples vary from -146 to -120‰ and -28,6 to -28,0‰, Egyptian samples show -130‰ to -100‰ and -29,5 to -28,2‰ for hydrogen and carbon isotopes, respectively. Sesame oil from Paraguay show values from -158 to -154‰ and -28,5 to -28,0‰, Nicaragua values from -176 to -174‰ and -29,7 to -28,6‰, El Salvador δ^2^H values around -169 and δ^13^C ratios from -29,6 to -28,7‰, Bolivia values from -215 to -194‰ and -30,7 to -30,0‰, one Senegalese sample a δ^2^H value of -172‰ and a δ^13^C value of -29,6‰, one sample from Mozambique values of -158 ‰ and -26,5‰ for hydrogen and carbon, respectively, one Ethiopian sample -136 ‰ and -27,5‰ for hydrogen and carbon, respectively and one sample from Turkey gives—109‰ and -27,6‰, for hydrogen and carbon isotopes, respectively.

Carbon isotopes of sesame oil from dark seeds from Egypt range from -29,5 to -29,1‰ VPDB, dark seeds samples from Nicaragua, El Salvador and Bolivia give -29,7, -29,6 and -30,7‰, respectively, always significantly lower than the corresponding oil from white seeds (Table 1).

**Fig 1:**
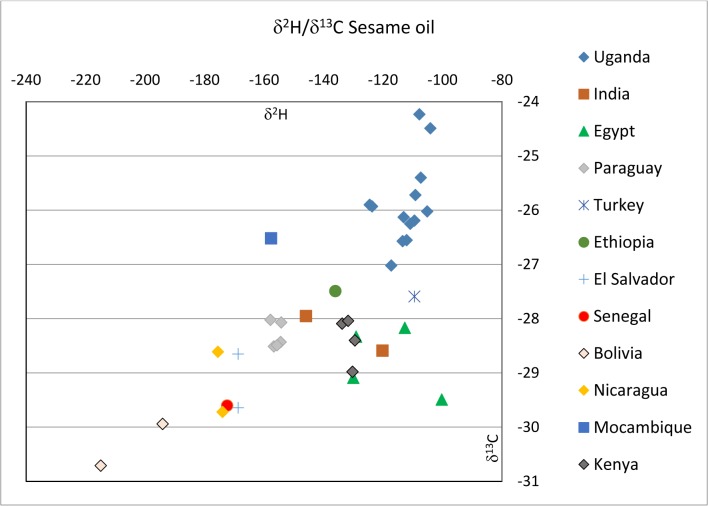
Isotope diagram showing C/H isotope ratios of the investigated sesame oil samples.

### Molecular markers

Using the cp DNA marker ccmp2, three different haplotypes could be identified (A = 202bp, B = 203bp, C = 203bp), with B being the most frequent haplotype, present in 88,9% of the samples (data not shown), whereas A and C being present in 19,4 and 2,8% of the samples respectively. 8,3% of the samples showed more than one haplotype per bulked sample, 2 having a combination of AB (161151, Nicaragua; Local (158x6022-1)-2-1, Uganda), one having a combination of BC (sample 00019 from Kenya). Due to the limited variation available in the investigated region of the chloroplast no spatial geographic variation could be deduced from the cpDNA data set.

The 9 different SSR marker regions showed different numbers of alleles per locus, ranging from 2 in BU668768 to 14 alleles in GBssr_sa_123 with allelic frequencies from 95 down to 1% depending on the locus. SSR markers from non-coding nuclear regions showed higher allele number than those developed based on ESTs.

**Fig 2:**
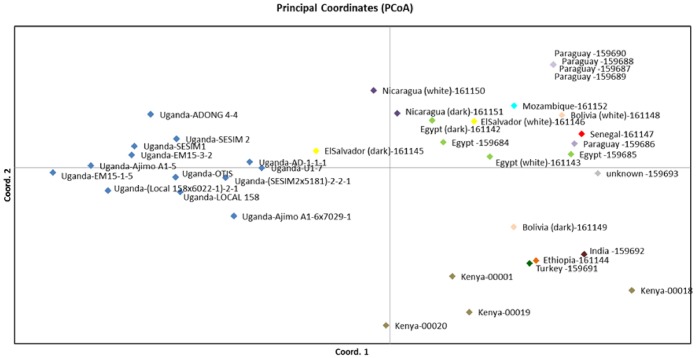
PCA analysis of the genetic differences between 38 bulked sesame samples from Europe, Africa, Asia and Latin America

Based on the nuclear DNA diversity results, 67 loci could be used in a pseudo dominant allele count using a digitalized format (0/1 coding) to conduct a principle component analysis. The 3 first components of Eigen vectors and Eigen values could explain 34,9% of the variation where the first component explained 15,9% and component 2 10,7% of the variation. This PCA analysis using the two most informative components one and two, revealed 3 genetically different clusters of sesame accessions, where samples from Kenya, India, Ethiopia and Turkey formed one cluster, samples from Uganda formed a distinct cluster with one El Salvador sample more close to Uganda. All the rest of the samples are falling into one big cluster (Fig 2) comprising all seed material acquired from traders from the international food market. Only one sample from Turkey, one from India as well as one sample from Ethiopia fall into the cluster of samples from ICRISAT/Kenya. Using nuclear SSRs, no genetic variation between black and white seeded samples could be observed. All of the different accessions showed a varying degree of diversity among samples from the same region. Four of the five samples from Paraguay had a 100% identical genetic profile in all the analysed loci.

We performed statistical analysis in order to analyse information content of combined SSR and isotopic data sets. The null hypothesis of no differences between *a priori* defined groups (i.e. assuming no constraints, as for the PCoA) was investigated using the PERMANOVA approach [46] implemented in vegan R package and applied to the distance matrixes of SSR (genetic distance) and isotope-based (eucledian distance) dataset, separately. The pseudo-F value shows (p<0.0001) that the patterns of samples are highly associated with the origin category, in both datasets. Then, a comparison between SSR and isotope-based datasets was carried out by means of procrustes analysis (PROTEST). PROTEST was performed on PCoA ordinations in order to evaluate the significance of the assessment of the distributions originating from SSR and isotopes [47]. The m12 value is related to the sum of the distances between each sample and its own counterpart in the other dataset. A m12 value of ~0.7 (p = 0.0001) shows no association.

A confirmatory Mantel test was also applied to the same distance matrices used for calculating the above mentioned PCoA ordinations. The Pearson correlation coefficient reported no correlation (r = 0.15, p<0.05).

## Discussion

The results for carbon and hydrogen isotopes (Fig 1) show a positive correlation of these two parameters and evidence a general interrelation between temperature and water availability. Tropical sesame oil samples from Uganda are most enriched in ^13^C and ^2^H, samples from Bolivia grown at high altitude (lower temperature) and at distance from the sea are most depleted. Stable isotope data of the seed material from ICRISAT/Kenya (0019, 0018, 0001 and 0020) show values distinctively lower in heavy isotope concentrations of both elements (^13^C and ^2^H) with respect to the Ugandan samples, as they were grown at a different location (Fig 1). The isotopic differences are thus to be explained by the different environmental conditions in Uganda and Kenya and give evidence that the samples have been harvested in Kenya and were transferred to Uganda.

DNA based investigations have identified significant genetic differences between the sesame seeds from Kenya as compared to Uganda and prove to be another potent tool to discriminate between geographic origins due to variations in the genetic pool. Regional geographic differentiation especially of land races revealed by DNA markers as detected in the Ugandan samples work well as long as seed and plant material is not transferred from one region to another. On the other hand—as can be seen with the Kenyan samples which cluster together with samples from Turkey, Ethiopia and India—whenever genetic material is transferred to new regions and integrated in international breeding programs at ICRISAT, the DNA based marker systems have their limitations.

The problem of having to analyze bulked seeds and thus having many alleles present per locus was solved by treating the SSR patterns as dominant marker type in genetic analysis, as in some bulked samples up to 8 different alleles were present. This way of analyzing the SSR pattern in bulked seeds might lead to an underestimation of rare alleles present in one sample due to the weak sampling power of the respective region in case of unbalanced mixtures. Wherever possible, single seed analysis should be preferred over bulked DNA analysis, as genetic analysis will be more informative on the single seed bases. Still, results show that the samples analyzed as bulked material from Turkey, India and Ethiopia cluster with the samples from Kenya, which were analyzed on a single seed bases, which indicates that there is no bias due to the type of investigated sample. Also the fact that the samples from Uganda and Kenya, both being analyzed on single seed level, do not cluster together, is an indication that there is no bias stemming from the molecular analysis method.

In regions with intense trade connections, mixing of local gene pools with gene pools from international breeding programs or planting of seed material from international seed dealers might be one reason why, other than with central African samples, material from different geographic origins clusters together when using DNA based analysis technologies. Still in all the 38 investigated samples moderate to high genetic variation is detected, except in the case of four of the five samples from Paraguay which exhibit an identical genetic fingerprint in all invested genomic regions. It is very unlikely that in an outcrossing species like sesame, where investigations have shown a degree of outcrossing of 4–62% (data not shown), planted material is genetically quite uniform, without introgression from neighboring fields. Possible explanations are that four of the five samples from Paraguay originate from the same field, or the same genotype is grown in vast production regions. As the isotope values of the Paraguay sesame seeds are all very similar, the first of the two explanations seems most likely, although also the possibility of production of genetically identical sesame plants under homogeneous conditions within a larger production region cannot be excluded.

A very important issue is the authenticity of the investigated samples. As we received the samples mainly from producers and production cooperatives marketing their products at food fairs, there is no direct proof of authenticity of geographic origin. However, as these persons and institutions want to sell their goods, most likely to our understanding, they will advertise their own products and none they will need to buy somewhere themselves. Additionally, as very often we received two or more products from these producers (e.g. black, beige and white sesame seeds), we have a control, if these different products from the same producer show similar isotope results, which would be rather unlikely if these products were just bought from somewhere. This homogeneity of results is observed (see Fig 1 and Table 1) in the results (especially δ^2^H) of the samples from Nicaragua, El Salvador, Paraguay, Egypt (2 producers), Kenya and Uganda (with the samples of the latter two countries being of verified origin anyway). It is not found in the samples from India (2 different producers) and Bolivia. Still, for the samples of declared Bolivian origin the authenticity is plausible due to the very special/low isotope values, which can be explained by growth in a high-altitude region.

The isotope values of the Egyptian samples are of interest, as they plot besides the general trend by showing distinctively lower carbon isotope values. This is most plausibly explained by intense irrigation preventing enrichment in ^13^C by closing of leaf stomata due to drought stress, as explained above. On the other hand the Mozambiquean sample shows an enriched carbon isotope value, probably caused by intense drought stress. The sesame oil sample from Turkey has a similar isotope signature as the Egyptian samples. This is remarkable as Turkey is located significantly further north and precipitation should thus be less enriched than samples of tropical and subtropical origins. Therefore this datum needs to be verified, as there is the possibility of incorrect declaration of geographic origin.

Sesame seed oil from dark sesame seeds has lower δ^13^C values by 0,8 to 1,3‰. There is little difference between oil from light and dark sesame seeds in the hydrogen isotope ratio, providing evidence that corresponding white and black sesame seed oils have been produced from seeds grown at the same locality, except for the Bolivian samples that must have been grown at different localities, due to the difference in hydrogen isotopes. These results indicate that black sesame seed plants might be to some extent less sensitive to drought stress (or better adapted) than white sesame plants, which might be a useful information for the selection and breeding of the optimal cultivar of sesame for a region with limited water resources. Using nuclear SSRs and cpSSRs, no significant genetic variation between black and white seeded samples could be observed. This might be attributed to the fact that the used nuclear SSR loci either lie in non-coding regions or in gene regions not relevant for the inheritance of seed coat colour. Studies on seed colour of sesame [37, 38] describe the inheritance of seed colour as a multigenic trait with little environmental influence, like in other species [39, 40].

The hydrogen and carbon isotope analyses and molecular markers of sesame of different origin are presented in this study. The isotope parameters are positively correlated and thus confirm the dominant influence of climate and water availability/drought stress on the investigated element isotope pattern. There exists a generally good discrimination between different origins; especially most of the investigated big producers (Uganda, India, Ethiopia, and Egypt) can be separated. Also the molecular markers prove to be a very potent tool for discrimination of origins. However, in routine analysis of commercial samples the almost global transfer and exchange of seeds and seedling material will blur the picture and reduce the power of this method. Furthermore, for a routine control of declared origin of sesame seeds a database of results from authentic samples from various regions and countries and several harvests will be needed, thus our study is a first promising step. Both methods differentiated the Kenyan sesame samples provided by the Ugandan partner from the seeds grown in Ugandan as a nice example for their complementarity. Some samples show offsets from the general trend line evidencing excellent or rather disadvantageous growing conditions. Additional analyses (e.g. fatty acids (FA) spectrum and FA-isotope composition and investigation of regional genetic differences) might help to further discriminate samples of different geographic origin. Sesame oil from black sesame seeds is more depleted in ^13^C with respect to sesame oil from white sesame seed from the identical locality. This indicates a better water use efficiency of black sesame plants.

The analytical comparison of stable isotopes and DNA based markers show the complementarity of both methods. Whereas the stable isotope results allow for a geographic differentiation and give a rough indication of physiological reactions to drought, the molecular markers distinguish genotypes based on genetic variation present in the seed lots, allowing the reconstruction of genetic relations between cultivars without considering the respective cropping region. Statistical analysis reveals that despite isotope- and DNA- datasets are associated with the “origin” category, they’re not depicting the same scenario.

## References

[1] Oplinger ES, Putnam DH, Kaminski AR, Hanson CV, Oelke EA, Schulte EE, et al. Sesame, Alternative Field Crops Manual. 1997. Available from: http://www.hort.purdue.edu/newcrop/afcm/sesame.html

[2] Chung C, Boyer T, Han S. Valuing Quality Attributes and Country of Origin in the Korean Beef Market. Journal of Agricultural Economics. 2009;60: 682–698.

[3] Bae SK, Lee KT. Discrimination of Genuine Sesame Oil from Imitations in the Consumer Market. Journal of the Korean Society of Food Preservation. 2009;16: 594–598

[4] Son HJ, Kang JH, Hong EJ, Lim CL, Choi JY, Noh BS. Authentication of Sesame Oil with Addition of Perilla Oil Using Electronic Nose Based on Mass Spectrometry. Journal of the Korean Society of Food Science and Technology. 2009;41: 609–614

[5] Yang YS, Kim JP, Seo KW, Cho BS, Gang GL, Kim ES, et al A Survey on Adulteration and Safety of Sesame oil Circulated in Gwangju. Journal of the Korean Society of Food Hygiene and Safety. 2008;23: 212–217

[6] Spangenberg JE, Ogrinc N. Authentication of vegetable oils by bulk and molecular carbon isotope analyses—with emphasis on olive oil and pumpkin seed oil. Journal of Agricultural and Food Chemistry. 2001;49: 1534–1540. 11312892 10.1021/jf001291y

[7] Hai Z, Wang J. Detection of adulteration in camellia seed oil and sesame oil using an electronic nose. Eur. J. Lipid Sci. Technol. 2006;108: 116–124.

[8] Seo H, Ha J, Shin DB, Shim SL, No KM, Kim KS, et al Detection of Corn Oil in Adulterated Sesame Oil by Chromatography and Carbon Isotope Analysis. J Am Oil Chem Soc. 2010;87: 621–626.

[9] Guo LX, Xu XM, Yuan JP, Wu CF, Wang JH. Characterization and Authentication of Significant Chinese Edible Oilseed Oils by Stable Carbon Isotope Analysis. J Am Oil Chem Soc. 2010;87: 839–848.

[10] Christoph N, Baratossy G, Kubanovic V, Kozina B, Rossmann A, Schlicht C, et al Possibilities and limitations of wine authentication using stable isotope ratio analysis and traceability. Part 2: Wines from Hungary, Croatia, and other European countries. Mitteilungen Klosterneuburg. 2004;54: 155–169.

[11] Camin F, Bontempo L, Heinrich K, Horacek M, Kelly S D, Schicht C, et al Multi-element (H, C, N, S) stable isotope characteristics of lamb meat from different European regions. Analytical and Bioanalytical Chemistry. 2007;389: 309–320. 17492274 10.1007/s00216-007-1302-3

[12] Schellenberg A, Chmielus S, Schlicht C, Camin F, Perini M, Bontempo L, et al Multielement Stable Isotope Ratios (H, C, N, S) of honey from different European regions. Food Chemistry. 2010;121: 770–777.

[13] Goitom Asfaha D, Quetel CR, Thomas F, Horacek M, Wimmer B, Heiss G, et al Combining isotopic signatures of N(87Sr)/(86Sr) and light stable elements (C, N, O, S) with multi-elemental profiling for the authentication of provenance of European cereal samples. Journal of Cereal Sciences. 2011;53: 170–177.

[14] Longobardi F, Casiello G, Sacco D, Tedone L, Sacco A. Characterisation of the geographical origin of Italian potatoes, based on stable isotope and volatile compound analyses. Food Chemistry. 2011;124: 1708–1713. 21740782

[15] Rummel S, Hölzl S, Horn P, Roßmann A, Schlicht C. The combination of stable isotope abundance ratios of H, C, N and S with 87Sr/86Sr for geographical origin assignment of orange juices. Food Chemistry, 2010;118: 890–900.

[16] Bontempo L, Camin F, Larcher R, Nicolini G, Perrini M, Roßmann A. Coast and year effect on H, O and C stable isotope ratios of Tyrrhenian and Adriatic italian olive oils. Rapid Commun. Mass Spectrom. 2009;23: 1043–1048. 10.1002/rcm.3968 19253913

[17] Iacumin P, Bernini L, Boschetti T. Climatic factors influencing the isotope composition of Italian olive oils and geographic characterisation. Rapid Commun. Mass Spectrom. 2009;23: 448 10.1002/rcm.3896 19125426

[18] Camin F, Larcher R, Nicolini G, Bontempo L, Bertoldi D, Perini M, et al Isotopic and elemental data for tracing the origin of European olive oils. Journal of Agricultural and Food Chemistry. 2010;58: 570–577. 10.1021/jf902814s 20000737

[19] Pasqualone A, Montemurro C, Caponio F, Blanco A. Identification of Virgin Olive Oil from Different Cultivars by Analysis of DNA Microsatellites. Journal of Agricultural and Food Chemistry. 2004;52: 1068–1071 14995099 10.1021/jf0348424

[20] Consolandi C, Palmieri L, Severgnini M, Maestri E, Marmiroli N, Agrimonti C, et al A procedure for olive oil traceability and authenticity: DNA extraction, multiplex PCR and LDR–universal array. Eur Food Res Technol. 2008;227: 1429–1438; 10.1007/s00217-008-0863-5

[21] Suits NS, Denning AS, Berry JA, Still CJ, Miller JB, Baker IT. Simulation of carbon isotope discrimination of the terrestrial biosphere. Global Biogeochemical Cycles. 2005;19: B1017–B1017.

[22] Ballantyne AP, Miller JB, Baker IT, Tans PP, White JWC. Novel applications of carbon isotopes in atmospheric CO_2_: what can atmospheric measurements teach us about processes in the biosphere? Biogeosciences. 2011;8: 3093–3106

[23] Krull E, Sachse D, Muegler I, Thiele A, Gleixner G. Compound-specific delta(13)C and delta(2)H analyses of plant and soil organic matter: A preliminary assessment of the effects of vegetation change on ecosystem hydrology. Soil Biology & Biochemistry. 2006;38: 3211–3221.

[24] Bowen GJ, Revenaugh J. Interpolating the isotopic composition of modern meteoric precipitation. Water Resources Research. 2003;39: 1299–1312.

[25] Nielsen LR, Kjær ED. Tracing timber from forest to consumer with DNA markers Danish Ministry of the Environment, Forest and Nature Agency 2008 http://www.skovognatur.dk/udgivelser.

[26] Tnah LH, Lee SL, Ng KKS, Tani N, Bhassu S, Othman RY. Geographical traceability of an important tropical timber (Neobalanocarpus heimii) inferred from chloroplast DNA. Forest Ecology and Management. 2009;258: 1918–1923

[27] Hormaza JI. Molecular characterization and similarity relationships among apricot (Prunus armeniaca L.) genotypes using simple sequence repeats. TAG Theoretical and Applied Genetics. 2002;104: 321–328 12582704 10.1007/s001220100684

[28] Gailing O, Wachter H, Heyder J, Schmitt HP, Finkeldey R. Chloroplast DNA analysis in oak stands (Quercus robur L.) in North Rhine-Westphalia with presumably Slavonian origin: Is there an association between geographic origin and bud phenology? Journal of Applied Botany and Food Quality. 2007;81: 165–171.

[29] Abdellatef E, Sirelkhatem R, Mohamed Ahmed MM, Radwan KH, Khalafalla MM. Study of genetic diversity in Sudanese sesame (Sesamum indicum L.) germplasm using random amplified polymorphic DNA (RAPD) markers. African Journal of Biotechnology. 2008;7: 4423–4427

[30] Bisht IS, Mahajan RK, Loknathan TR, Agrawal RC. Diversity in Indian sesame collection and stratification of germplasm accessions in different diversity groups. Genetic Resources and Crop Evolution. 1998;45: 325–335, 10.1023/A:1008652420477

[31] Ercan G, Taskin M, Turgut K. Analysis of genetic diversity in Turkish sesame (Sesamum indicum L.) populations using RAPD markers. Genetic Resources and Crop Evolution. 2004;51: 599–607, 10.1023/B:GRES.0000024651.45623.f2

[32] Pham TD, Bui TM, Werlemark G, Bui TC, Merker A, Carlsson AS. A study of genetic diversity of sesame (Sesamum indicum L.) in Vietnam and Cambodia estimated by RAPD markers. Genetic Resources and Crop Evolution. 2009;56: 679–690

[33] Dixit A, Jin MH, Chung JW, Yu JW, Chung HK, Ma KH, et al Development of polymorphic microsatellite markers in sesame (Sesamum indicum L.) Genes & Genomes. 2005;5: 736–738

[34] Cho YI, Park JH, Lee CW, Ra WH, Chung JW, Lee JR, et al Evaluation of the genetic diversity and population structure of sesame (Sesamum indicum L.) using microsatellite markers.Genes & Genomics. 2011;33: 187–195,

[35] Horacek M, Min JS. Discrimination of Korean beef from beef of other origin by stable isotope measurements. Food Chemistry. 2010;121: 517–520

[36] Wills DM, Hester ML, Liu A, Burke JM. Chloroplast SSR polymorphisms in the Compositae and the mode of organellar inheritance in Helianthus annuus. Theor Appl Genet. 2005;110: 941–947 10.1007/s00122-004-1914-3 15690173

[37] Shim KB, Lee YY, Cho SK, Pae SB, Suh DY. Inheritance of seed coat color in sesame. Korean Journal of Breeding. 2005;37: 1–4

[38] Falusi OA. Segregation of genes controlling seed colour in sesame (*Sesamum indicum* Linn. African Journal of Biotechnology. 2007;6: 2780–2783

[39] Lakshmi Padmaja K, Arumugam N, Gupta V, Mukhopadhyay A, Sodhi YS, Pental D, et al Mapping and tagging of seed coat colour and the identification of microsatellite markers for marker-assisted manipulation of the trait in Brassica juncea. TAG Theoretical and Applied Genetics. 2005;111: 8–14, 10.1007/s00122-005-1933-8 15902399

[40] Haughn G, Chaudhury A. Genetic analysis of seed coat development in Arabidopsis. Trends Plant Sci. 2005;10: 472–7. 16153880 10.1016/j.tplants.2005.08.005

[41] Jöbstl D, Bandoniene D, Meisel T, Chatzistathis S. Identification of the geographical origin of pumpkin seed oil by the use of rare earth elements and discriminant analysis. Food Chemistry. 2010;123: 1303–1309.

[42] Horacek M, Min JS, Heo SC, Soja G. Discrimination between ginseng from Korea and China by light stable isotope analysis. Analytica Chimica Acta. 2010;682: 77–81. 10.1016/j.aca.2010.09.046 21056718

[43] Schuelke M. An economic method for the fluorescent labelling of PCR fragments. Nature Biotechnology. 2000;18: 233–234 10657137 10.1038/72708

[44] Oksanen J, Blanchet FG, Kindt R, Legendre P, Minchin PR, O’Hara RB, et al. vegan: Community Ecology Package. 2014. Available from: http://cran.r-project.org/web/packages/vegan/index.html

[45] R Development Core Team. R: A language and environment for statistical computing (Vienna, Austria: R Foundation for Statistical Computing). 2009 Available from: http://CRAN.R-project.org/package=vegan, http://www.R-project.org

[46] Anderson MJ. A new method for non-parametric multivariate analysis of variance: NON-PARAMETRIC MANOVA FOR ECOLOGY. Austral Ecology. 2001;26: 32–46.

[47] Peres-Neto P, Jackson D. How well do multivariate data sets match? The advantages of a Procrustean superimposition approach over the Mantel test. Oecologia. 2001;129: 169–178. 28547594 10.1007/s004420100720

[48] Legendre P, Legendre L. Numerical Ecology. 2012;Elsevier, 1006 pages.

